# Survey of Long-Term Experiences of Sperm Cryopreservation in Oncological and Non-Oncological Patients: Usage and Reproductive Outcomes of a Large Monocentric Cohort

**DOI:** 10.3389/fonc.2021.772809

**Published:** 2021-11-05

**Authors:** Nadine Lackamp, Ina Wilkemeyer, Ivan Jelas, Ulrich Keller, Lars Bullinger, Sebastian Stintzing, Philipp le Coutre

**Affiliations:** ^1^ Department of Hematology, Oncology, and Tumor Immunology, Charité - Universitätsmedizin Berlin, corporate member of Freie Universität Berlin and Humboldt-Universität zu Berlin, Berlin, Germany; ^2^ Department of Urology, Charité - Universitätsmedizin Berlin, corporate member of Freie Universität Berlin and Humboldt-Universität zu Berlin, Berlin, Germany

**Keywords:** sperm cryopreservation, cancer, fertility preservation, assisted reproduction techniques (ART), chemotherapy, natural fertility, reproductive outcomes, cancer treatment

## Abstract

Progress in oncological treatment has led to an improved long-term survival of young male cancer patients over the last decades. However, standard cancer treatments frequently implicate fertility-damaging potential. Cryopreservation of sperm is the current standard option to preserve patient’s fertility after treatment, yet long-term data on usage and reproductive experiences is still limited. Natural fertility after treatment and especially in relation to the type of treatment has been poorly analyzed so far. Therefore, we performed a retrospective survey including male patients with an indication for gonadotoxic treatment who cryopreserved reproductive material at our institution between 1994 and 2017. Study questionnaires regarding treatment, material usage, and reproductive outcomes were sent to eligible patients. Additionally, semen analyses of study participants from the time of cryopreservation were evaluated. A total of 99 patients were included in the study. Respondents’ median age was 38.0 years. Most frequent diagnoses were testicular cancer (29.3%) and lymphoma (26.3%). A further 8.1% suffered from autoimmune diseases. Testicular cancer patients had a significantly lower pre-treatment median sperm concentration (18.0 million/ml) compared to non-testicular cancer patients (54.2 million/ml). Until November 2020, the determined sperm usage and cumulative live-birth rate per couple were 17.2% and 58.8%, respectively. Most sperm users received treatments with high (40.0%) or intermediate (33.3%) gonadotoxic potential. 20.7% of all patients reported to had fathered at least one naturally conceived child after treatment, this being the case especially if they had been treated with less or potentially gonadotoxic therapies. In conclusion, our findings emphasize the importance of sperm cryopreservation in the context of male fertility preservation. Furthermore, they indicate that the gonadotoxic potential of patients’ treatments could represent a predictive factor for sperm usage.

## Introduction

Due to advances in both oncological diagnosis and treatment, over the last few decades the long-term survival of young male cancer patients has significantly improved. However, standard cancer treatments – including radiation therapy, classical cytotoxic substances, and increasingly personalized, modern approaches – often implicate potential risks for patients’ future fertility.

Several studies have shown reduced semen quality in some cancer patients even prior to the initiation of their treatment. This impairment of spermatogenesis, induced by the malignancies themselves, especially applies to patients with testicular cancer, the most frequently diagnosed cancer type in young men (1–3). In addition, various cancer treatments cause damage to spermatogenesis: Firstly, cytotoxic agents frequently compromise spermatogenesis, at least temporarily, and in some cases even induce permanent azoospermia. Among the cytotoxic substances, most alkylating agents and cisplatin are known to be strongly gonadotoxic; however, their deleterious effects on spermatogenesis ultimately depend on drug combination and dosage (4, 5). Secondly, the irradiation of the radiosensitive testicles deteriorates their function in a dose dependent way. Doses above four Gy can cause permanent germ cell defects, and 16 – 20 Gy can lead to irreversible azoospermia. Although recovery of spermatogenesis is attainable, it is highly dependent on radiation doses and the number of cycles conducted, requiring up to five years or even longer. Leydig cells of adults were observed to be more resistant to radiotherapy, nevertheless, in some cases testosterone replacement therapy after gonadal radiation is still necessary (6). Thirdly, conditioning regimens for hematopoietic stem cell transplantations (HSCT) have an extremely high gonadotoxicity due to aggressive chemotherapies and/or total body irradiation (TBI) combinations (5, 7). Finally, fertility can be reduced after gonadal surgeries, such as the radical inguinal orchiectomy, which is the mainstay of treatment for testicular cancer patients. Several studies report adverse effects on semen parameters and hormonal functions after radical inguinal orchiectomies (6).

Patients’ fertility after cancer treatment is not predictable. Therefore, fertility preservation is a primary concern for cancer patients of reproductive age and should be discussed as early as possible before treatment is initiated. Currently, the standard option for fertility preservation in men is cryopreservation of ejaculated semen (8). The number of cancer patients who undergo sperm cryopreservation has increased during the last 20 years (1, 9). Regardless, in many countries, pre-treatment sperm banking has not been established in clinical practice or is underutilized when offered (2, 10, 11). Depending on the country, costs for storage differ (11, 12). In Germany, as of the beginning of July 2021, the costs of sperm storage for cancer patients who have to undergo fertility-damaging therapies are covered by the public health insurance, according to the decision of the Federal Joint Committee (G-BA).

Reported usage rates of stored material remain low, often less than 10%, with a slight increase when examining long-term follow-ups of above 10 years (1, 9, 13–18). Different variables impacting long-term usage of sperm have been described so far, including age at the time of cryopreservation, type of cancer, and total number of cryopreserved spermatozoa (18). Nevertheless, many influencing factors are still unexplored. After deciding to use the frozen semen, couples proceed with assisted reproductive techniques (ART) and achieve parenthood in 35 – 80% of cases (1, 14, 19, 20).

Ample literature on outcomes of sperm cryopreservation in cancer patients exists, however, various aspects have not yet been analyzed sufficiently. Data on long-term follow-ups is still limited and more research is needed to make reliable conclusions about ART usage and success rates (1, 9, 11, 13–15, 19, 20). Moreover, studies exploring the relation between different cancer treatment types, sperm usage, and reproductive outcomes of natural fertility after treatment are lacking (19, 21–24). In addition, fertility preservation also concerns young patients affected by various non-oncological medical conditions. One group in this context are patients suffering from autoimmune diseases treated with gonadotoxic immunotherapy or HSCT. To our knowledge, cryopreservation experiences and outcomes from non-oncological patients who undergo fertility-damaging treatments have been barely reported (25).

In order to address the above-mentioned aspects of interest, and to add new findings to the existing literature, we performed a questionnaire-based survey of patients who cryopreserved reproductive material at our institution over a period of 22 years. This study aimed to analyze participants’ usage of stored material, resulting ART outcomes, and natural reproduction outcomes after treatment, and to examine these outcomes with regard to treatment types and their gonadotoxic potential.

## Materials and Methods

### Availability of Materials

The study questionnaire and original data underlying the conclusions listed in the manuscript are included in the supplementary material; further inquiries can be directed to the corresponding author.

### Study Population and Data Source

The study population included all male patients who cryopreserved reproductive material at the Cryobank of Charité – Universitätsmedizin Berlin in the period from February 1994 until November 2017. All oncological and non-oncological patients with diagnoses requiring potentially gonadotoxic treatments were considered. Inclusion criteria were an age above 18 years, patient data available in our patient database and a German contact address. Deceased patients as well as patients with unsuccessful cryopreservation were excluded.

A study-specific questionnaire was developed by the researchers including the following questions: sociodemographic characteristics (personal data, occupation), questions related to diagnosis and treatment (disease/treatment as indication for cryopreservation, type of cancer/other diagnosis, date of initial diagnosis, type/s of treatment, date/s of treatment, relapses of disease, current disease status), questions concerning the cryopreservation (date/s, frequency, indication, time of cryopreservation in relation to treatment), questions about usage of stored material (material request, collection date, usage for ART, successful live-births, unsuccessful ART procedures, usage planned in future), and questions regarding family and children [marital status, number of children, characteristics of children (biological/not biological, type of reproduction, date of birth, sex, birth weight, height at birth, events during birth, current health status, date of birth of mother)].

### Procedure and Data Management

Our patient database was reviewed to find potential study participants. Out of 1089 patients, who were listed in the Cryobank for the mentioned period, 774 were identified as possible participants. In June and September 2020, the eligible patients were sent a study pack containing a participant’s information sheet, a consent form and copy, the study questionnaire and a pre-paid envelope addressed to the director of the study. 259 study packs could not be delivered due to outdated mailing addresses. 515 study packs could be delivered. A total of 111 questionnaires were completed and returned, yielding a response rate of 21.5%. Two respondents were relatives of recently deceased patients and had completed the questionnaires for them. Their data were excluded from the analysis. Further 10 patients were excluded because of missing indication for gonadotoxic treatment.

### Semen Analyses

Semen analyses at the time of cryopreservation were requested from our Cryobank for patients included in the study. 82 analyses of ejaculated semen and three analyses of testicular tissue could be provided. The researchers evaluated sperm concentration, progressive and total motility (progressive and non-progressive motility) based on reference parameters from the World Health Organization (WHO) guidelines of 1999 and 2010, respectively (26, 27).

### Data Analysis

Data was entered into a password protected secure database. Not all respondents replied to all questions. Where possible, missing, or inconsistent data was revised and completed with data from our patient database. The number of patients whose data was still missing after this procedure is indicated and the respective patients were excluded from percentage calculations. Missing day or month specifications were replaced with the first day of a month or the first month of a year, respectively.

Data was analyzed in IBM SPSS Statistics Version 27. Categorical variables were presented as percentages. Continuous variables were presented as medians and interquartile range (IQR). Since the variables did not follow normal distribution, non-parametric tests were used to compare patient groups (Mann-Whitney-U-test and Chi-square-test). A p-value <0.05 was considered as statistically significant. Diagnosis groups were subsumed into appropriate upper-level groups for statistical testing.

Due to the retrospective design of the study, randomization or blinding of participants were not applicable. No power calculation was performed as the sample size directly resulted from the number of respondents to the questionnaire.

## Results

### Characteristics and Treatments of Respondents

**Figure 1:**
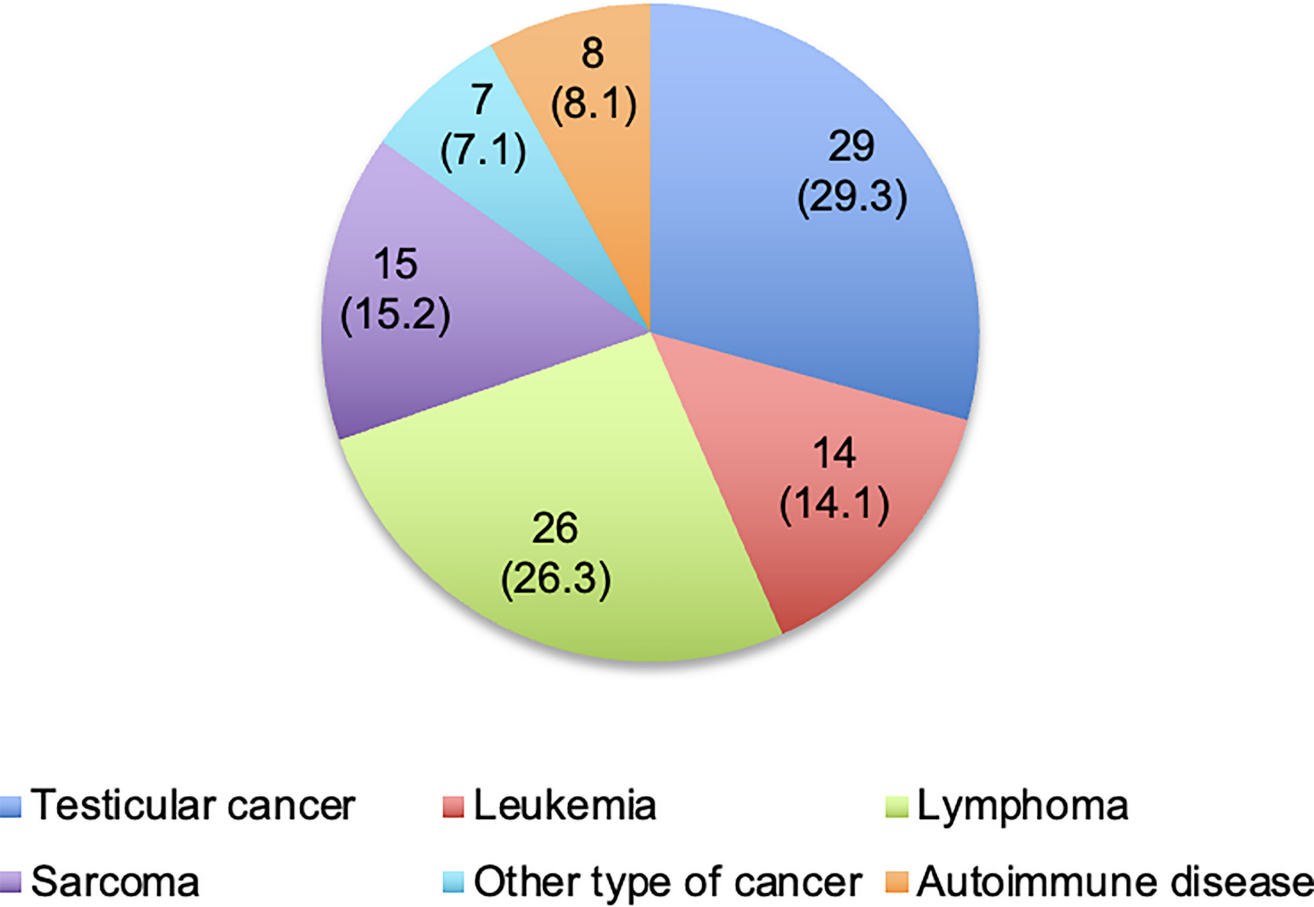
Diagnoses of respondents. Values are presented as number of patients (%). One patient with other type of cancer was additionally diagnosed with testicular cancer during the follow-up.

A total of 99 patients were included in the study. 91 patients (91.9%) suffered from oncological and 8 patients (8.1%) from non-oncological diseases. Most frequent diagnoses were testicular cancer (29.3%) and malignancies of the lymphatic and hematopoietic tissue (40.4%), as illustrated in **Figure 1**. All non-oncological patients were diagnosed with autoimmune diseases (namely membranous glomerulonephritis (two patients), neurosarcoidosis, Crohn’s disease, autoimmune thrombocytopenia, central nervous system vasculitis, systemic lupus erythematosus and septic granulomatosis). Respondents’ median age at the time of the survey was 38.0 years (IQR: 31.0 – 46.0 years). Further characteristics of the participants are shown in **Table 1**.

**Table 1:** Respondents’ characteristics.

Characteristics	
Age at time of survey, median (IQR)	38.0 (31.0 – 46.0)
Age at diagnosis, median (IQR)	27.0 (20.0 – 36.0)
Age at cryopreservation, median (IQR)	29.0 (20.0 – 36.0)
Marital status, n (%)	
Single	32 (33.7)
Married	45 (47.4)
In Partnership	16 (16.8)
Widowed	1 (1.1)
Other	1 (1.1)
N.a.	4
Reproductive material stored, n (%)	
Ejaculated semen	82 (96.5)
Testicular tissue	3 (3.5)
N.a.	14
Time of cryopreservation, n (%)	
Before treatment*	94 (94.9)
After treatment initialization	5 (5.1)

*One of the patients did not undergo the planned gonadotoxic treatment.

**Figure 2:**
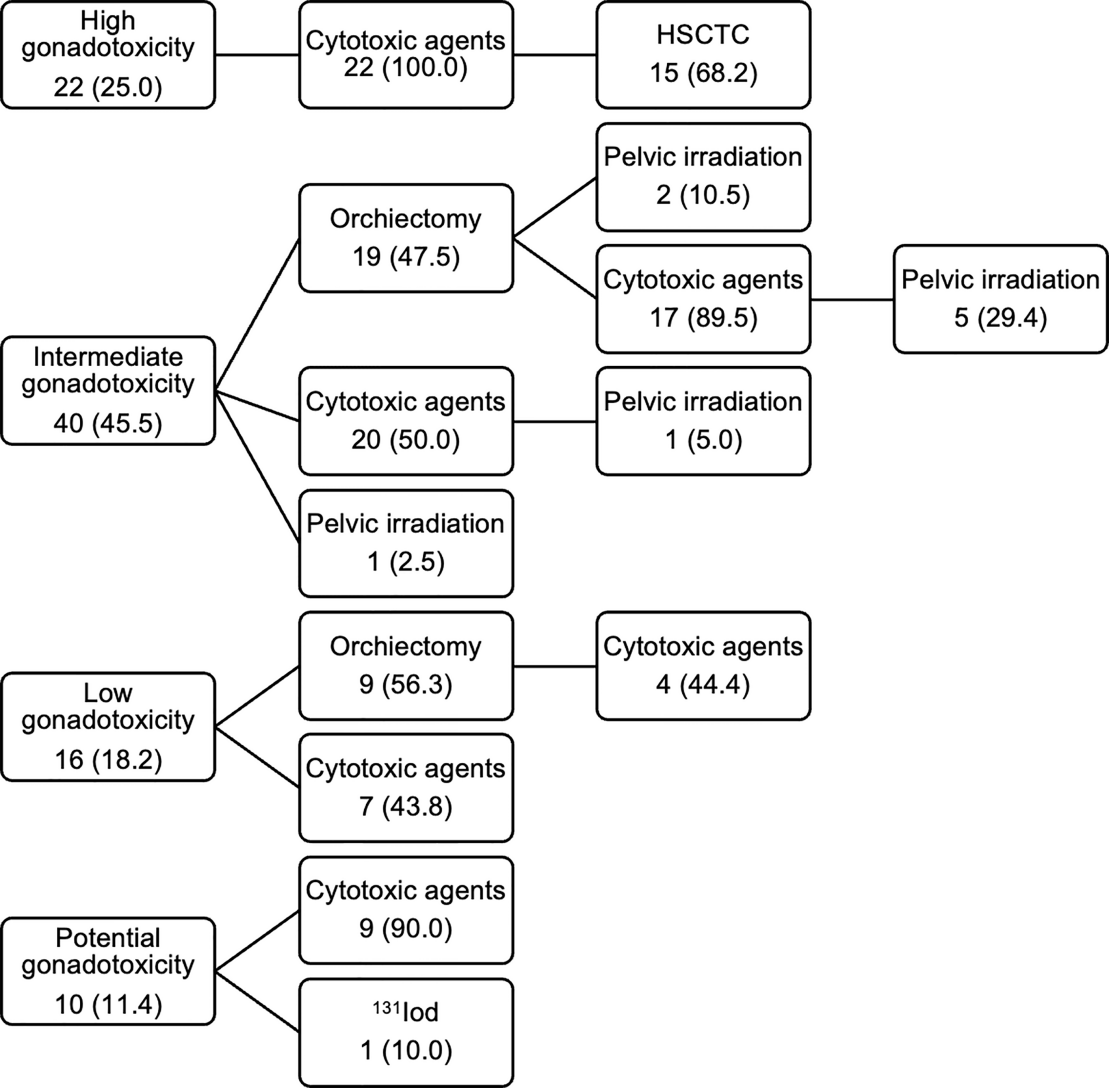
Treatment categories and treatment types of respondents. Values are presented as number of patients (%). HSCTC, HSCT conditioning regimen.

The participants received one (60.2%) or multiple (39.8%) of the following gonadotoxic treatment types: cytotoxic agents (chemotherapy or immunotherapy), orchiectomy, radiotherapy, and/or HSCT. Radiation therapy was included as a fertility-damaging treatment exclusively in case of gonadal irradiation or TBI as a conditioning regimen for HSCT, meaning that irradiation of other body parts (13 patients) or unknown areas (9 patients) were not included. One patient did not undergo the planned treatment and was excluded from treatment analyses. The fertility-damaging treatments were classified into four categories depending on their gonadotoxic potential (**Table 2**). In total, 89 patients (90.8%) were treated with cytotoxic agents as part of their therapy regimens. 10 patients did not specify the cytotoxic substances and were excluded from the treatment classification. **Figure 2** illustrates the distribution of the treatment categories in the study population.

**Table 2:** Treatment categories according to the gonadotoxic potential.

Treatment category	Treatment or cytotoxic agent
Category 1: High gonadotoxicity	HSCT conditioning (chemotherapy and/or TBI), BEACOPP, VAC
Category 2: Intermediate gonadotoxicity/risk for prolonged azoospermia	Pelvic irradiation, cyclophosphamide, cisplatin, ifosfamide, lomustine, BEP, VIP, VIDE
Category 3: Low gonadotoxicity/risk for only temporary reduction in sperm counts	Radical inguinal orchiectomy, vincristine, vinblastine, carboplatin, doxorubicin, bleomycin, methotrexate, azathioprine, ABVD, CHOP
Category 4: Potential gonadotoxicity (existing data not sufficient)	Rituximab, bendamustine, imatinib, cladribine, ^131^Iod

Data from Poorvu et al. (28), additionally from Meistrich et al. (4), Vakalopoulos et al. (29), Traila et al. (30), and Zang et al. (31).

BEACOPP, bleomycin, etoposide, doxorubicin, cyclophosphamide, vincristine, procarbazine, prednisone; VAC, vincristine, actinomycin D, cyclophosphamide; BEP, bleomycin, etoposide, cisplatin; VIP, cisplatin, etoposide, ifosfamide; VIDE, vincristine, ifosfamide, doxorubicin, etoposide; CHOP, cyclophosphamide, doxorubicin, vincristine, prednisone.

Five patients (5.1%) underwent cryopreservation after treatment initialization or partial treatment completion. Among them, three patients had been orchiectomized and two patients had been treated with chemotherapy prior to cryopreservation.

### Semen Analyses

Semen analyses of ejaculated sperm at the time of cryopreservation were available for 82 of 99 patients. Median sperm concentration [37.9 million/ml (IQR: 17.3 – 93.2 million/ml)], progressive [47.0% (IQR: 32.0 – 55.0%)] and total sperm motility [56.0% (IQR: 43.3 – 65.0%)] of patients who cryopreserved sperm before initiating gonadotoxic therapy were normal. The five patients who stored material after partial treatment completion had a median sperm concentration of 20.5 million/ml (IQR: 13.2 – 56.6 million/ml), progressive motility of 32.0% (IQR: 24.0 – 55.0%) and total motility of 38.0% (IQR: 35.0 – 63.5%).

Pre-treatment semen analyses according to diagnosis groups are shown in **Table 3**. Testicular cancer patients had a significantly lower median sperm concentration [18.0 million/ml (IQR: 9.7 – 37.8 million/ml)] compared to non-testicular cancer patients (54.2 million/ml (IQR: 23.0 – 110.2 million/ml), Mann-Whitney-U-test, p = 0.003). No significant differences in progressive and total motility between the two groups were observed (Mann-Whitney-U-test, p = 0.656 and p = 0.688).

**Table 3:** Pre-treatment semen characteristics at the time of cryopreservation according to diagnosis groups.

Diagnosis group	Sperm concentration (10^6^/ml)	Progressive motility (%)	Total motility (%)
Testicular cancer	18.0 (9.7 – 37.8)	48.0 (23.0 – 55.0)	56.0 (35.0 – 65.0)
Non-testicular cancer	54.2 (23.0 – 110.2)	46.0 (32.0 – 55.8)	56.0 (44.0 – 65.5)
Leukemia	43.0 (22.7 – 118.2)	44.0 (33.0 – 54.0)	54.0 (47.0 – 64.0)
Lymphoma	33.0 (14.0 – 91.8)	50.0 (30.0 – 54.8)	56.5 (39.8 – 66.0)
Sarcoma	58.8 (19.1 – 171.0)	45.0 (31.5 – 50.5)	55.0 (42.5 – 63.5)
Other type of cancer	51.0 (22.7 – 138.0)	32.5 (28.0 – 59.5)	43.0 (30.0 – 62.0)
Autoimmune disease	97.6 (68.7 – 224.6)	61.5 (52.5 – 68.3)	66.5 (59.3 – 73.8)

Values are presented as median (IQR).

### Sperm Usage and ART Outcomes

Overall, 17 patients (17.2%) decided to use their cryopreserved material for ART procedures. In 13 cases, the reproductive material was ejaculated sperm, while the reproductive material remained unknown in the other four cases. The median age was 32.0 years (IQR: 27.0 – 36.5 years) at the time of cryopreservation. No significant difference from the median age at the time of cryopreservation of patients who did not use their material [27.0 years (IQR: 20.0 – 35.3 years)] was observed (Mann-Whitney-U-test, p = 0.135). **Table 4** summarizes the diagnosis groups and treatment categories of patient groups who used and did not use the cryopreserved material. All six sperm users in treatment category 1 (40.0%) were treated with highly sterilizing chemotherapy and three of them (20.0%) additionally underwent HSCT. Out of the five users in category 2 (33.3%), three (20.0%) were treated with orchiectomy and chemo-/chemoradiotherapy, and two (13.3%) exclusively with chemotherapy. The three sperm users in category 3 (20.0%) received less gonadotoxic agents and one of them (6.7%) had previously been orchiectomized. One sperm user (6.7%) underwent radioiodine therapy. No significant correlation between sperm usage and diagnosis group was observed (Chi-square-test, p = 0.991).

**Table 4:** Diagnosis groups and treatment categories of sperm users and non-users, and patients with and without naturally conceived children after gonadotoxic treatment.

Diagnosis group/treatment category	Use of cryopreserved sperm		Natural conception after treatment	
	Yes (n = 17)	No (n = 82)	Yes (n = 19)	No (n = 73)
*Diagnosis group*				
Testicular cancer	5 (29.4)	24 (29.3)	7 (36.8)	20 (27.4)
Non-testicular cancer	12 (70.6)	58 (70.7)	12 (63.2)	53 (72.6)
Leukemia	1 (5.9)	13 (15.9)	3 (15.8)	11 (15.1)
Lymphoma	6 (35.3)	20 (24.4)	5 (26.3)	19 (26.0)
Sarcoma	3 (17.6)	12 (14.6)	2 (10.5)	11 (15.1)
Other type of cancer	1 (5.9)	6 (7.3)		7 (9.6)
Autoimmune disease	1 (5.9)	7 (8.5)	2 (10.5)	5 (6.8)
*Treatment category*				
Category 1: High gonadotoxicity	6 (40.0)	16 (21.9)	2 (12.5)	18 (27.3)
Category 2: Intermediate gonadotoxicity	5 (33.3)	35 (47.9)	5 (31.3)	32 (48.5)
Category 3: Low gonadotoxicity	3 (20.0)	13 (17.8)	4 (25.0)	12 (18.2)
Category 4: Potential gonadotoxicity	1 (6.7)	9 (12.3)	5 (31.3)	4 (6.1)
Treatment not realized		1		1
N.a.	2	8	3	6

Values are presented as number of patients (%).

The median storage time until the first material request was 26.0 months (IQR: 16.5 – 65.5 months). Most of those who utilized the frozen material (13 patients, 76.5%) did so during the first five years of storage. The highest number of sperm usages was observed in the second year (six patients, 35.3%). During the first 10 years of storage, 94.1% of the users requested their frozen semen, and only one patient (5.9%) used his semen after a longer storage time. He was 17 years old at the time of cryopreservation. Among the non-users, 26 patients (34.7%) stated they would potentially make use of their material in the future. 10 sperm users (58.8%) achieved parenthood of at least one ART-conceived child. However, seven patients (41.2%) reported they had remained childless after unsuccessful ART procedures, yielding a cumulative live-birth rate per couple of 58.8%. All users had cryopreserved their semen prior to gonadotoxic treatment except for one testicular cancer patient, whose sperm cryopreservation was commissioned after orchiectomy. His ART procedures remained unsuccessful. Analysis of storage periods showed no significant difference in storage time between sperm users with successful [20.0 months (IQR: 17.3 – 42.5 months)] and unsuccessful ART outcomes [44.0 months (IQR: 15.0 – 116.0 months, Mann-Whitney-U-test, p = 0.261)]. The longest storage time before sperm usage resulting in fatherhood was 78 months.

A total of 20 children were born from ART cycles (8 singletons and six pairs of twins). Additionally, one patient stated he had an ART-conceived child from cryopreserved material that was not stored at our institution. The females’ median age at the time of childbirth was 35.0 years (IQR: 32.0 – 36.0 years). One ART-conceived child (4.8%) suffered from a pulmonary valve stenosis and one pair of twins (9.5%) was reported deceased at the time of the survey. All the other children (85.7%) were reported to be alive, healthy, and normally developing. Birth weights and heights at birth are tabulated in **Table 5** (percentiles were incalculable due to missing information on gestational age). Not all patients provided the appropriate data.

**Table 5:** Birth weights and heights of newborns.

Newborns	Birthweight (grams)	Height at birth (cm)
All newborns	3300.0 (2765.0 – 3660.0)	52.0 (49.0 – 53.0)
ART-conceived twins	1950.0 (1470.0 – 2330.0)	45.5 (41.5 – 47.5)
ART-conceived singletons	3050.0 (2882.5 – 3355.0)	52.0 (50.5 – 53.0)
Naturally conceived twins after treatment	1870.0	44.5
Naturally conceived singletons after treatment	3570.0 (3140.0 – 3910.0)	52.0 (50.0 – 54.0)

Values are presented as median (IQR).

### Natural Fertility of Respondents

In total, 92 patients provided detailed data on their biological children. 31 patients (33.8%) reported to have fathered at least one child from natural conception, 8 patients (8.7%) exclusively had ART-conceived children, and two patients (2.2%) had children of unspecified type of reproduction. One patient (1.1%) stated to have a pregnant partner at the time of the survey and 50 patients (54.3%) reported to have no biological children. Among those with naturally conceived children, 12 patients had achieved parenthood before (13.1%), 9 patients after (9.8%), and 10 patients (10.9%) before as well as after receiving gonadotoxic treatments. The first group included three sperm users, the second and third group one sperm user each. **Table 4** illustrates diagnosis groups and treatment categories of patient groups who fathered and did not father naturally conceived children after treatment. If available, birth weights and heights at birth of naturally conceived children after treatment are summarized in **Table 5** (percentiles were incalculable due to missing information on gestational age).

## Discussion

In the present survey we reported on experiences of sperm cryopreservation and outcomes of natural fertility in oncological and non-oncological patients at the Cryobank of Charité – Universitätsmedizin Berlin over a period of 22 years. Our paper supplements findings to the existing data on cryopreservation usage and success rates and is one of the first to analyze the relation between gonadotoxic treatment types and reproductive outcomes (19, 21–24).

### Integration of Study Findings in the Current State of Research

Our findings regarding patients’ characteristics and analysis of pre-treatment semen parameters are mainly in line with those presented by other authors. As already observed in previous studies, oncological patients in this survey were most frequently diagnosed with testicular cancer and tumors of the lymphatic and hematopoietic tissue (1, 9, 13, 15, 17–20). Although our analysis of pre-treatment semen parameters at the time of cryopreservation showed a huge interindividual variability, the lowest median sperm count was observed in patients with malignant testis tumors. Similar findings were reported by other authors (1, 5, 15, 17, 18, 20). Factors impairing spermatogenesis caused by testicular tumors even prior to treatment initiation seem to be multifactorial, including direct parenchymal damage and replacement by the malignancy, interferences in the hypothalamic-pituitary-gonadal axis, systemic inflammation, and increased oxidative stress (6).

The sperm usage rate in our study (17.2%) was higher compared to the 3 – 10% experienced by most of the other clinics (1, 9, 13–15, 20). For example, Ferrari and colleagues calculated in their review an aggregated usage rate of 8% (30 studies included) (16). However, a much higher usage rate of 27% was reported by Hammarberg and colleagues, who performed a cross-sectional survey (19). In contrast, the live-birth rate per couple we observed (58.8%) corresponds to findings reported in previous studies, even though the rates for each study vary strongly (35 – 80%) (1, 14, 19, 20). Nevertheless, when comparing the usage and ART success rates in our cohort to other results, the number of patients and a possible participation bias should be considered.

### Variables Affecting Sperm Usage

Although the usage rate in the present study was a bit higher compared to previous, it remained relatively low. In the following, we want to discuss on some variables that may affect the number of sperm usages:

Period of follow-up: Some authors suggested that sperm usage might increase with longer follow-up (16, 17, 32). However, our results did not confirm this (94.1% of the sperm users requested their material within the first 10 years of storage). Similarly, in their latest study, Ferrari and colleagues showed only a marginal increase in the usage rate (from 9.4% to 12.0%) by extending the period of follow-up beyond 10 years (18).

Type of gonadotoxic treatment: 40.0% of the sperm users compared to only 21.9% of the non-users in our cohort received highly sterilizing treatments. This observation indicates that treatments with strong gonadotoxicity might represent a predictive factor for future sperm usage even though our sample sizes were too small to apply reliable statistical tests to show the correlation. To our knowledge, the relation between different types of treatment and usage of cryopreserved sperm has not yet been analyzed sufficiently and, hence, further research is necessary to confirm our results.

Outcomes of natural reproduction: One of the reasons for low sperm usage rates could be maintained or recovered fertility. Given that 20.7% of our patients reported to have fathered children from natural conception after treatment, a substantial proportion of patients have retained or retrieved fertility. Nevertheless, two of them used their cryopreserved sperm. More research is needed to draw further conclusions on this aspect (19).

Costs for storage: Since, in some countries, the costs of cryopreserving reproductive material are born by patients themselves, they may affect cryopreservation rates and future sperm usage. However, no consistent data on this regard exists (11, 12). In Germany, since the beginning of July 2021, Public Health Insurance covers the costs for cryopreservation, specifically for male cancer patients under 50 years to preserve ejaculated sperm or testicular tissue when undergoing fertility-damaging therapies. Previously, such patients had to bear the costs privately and some may have chosen not to cryopreserve or to maintain the storage for this reason. It remains to be seen to what extent the alteration in insurance coverage will influence sperm cryopreservation, storage, and usage rates.

### Future Perspectives

The gonadotoxic potential of many commonly used chemotherapeutics is still not definitely explored. Despite this, we want to raise the topic of modern, targeted therapies and their impact on the male reproductive system as an important direction for future research. The effects of targeted therapies on male fertility are poorly investigated, and some of those agents could be less damaging compared to classical chemo- and radiotherapy regimes. So far, studies exploring the harmful effects on spermatogenesis caused by the tyrosine kinase inhibitor imatinib have demonstrated controversial outcomes. Fertility impairments might be only modest in adult males or more severe when the agent is given during puberty (4). Since the use of targeted therapies in cancer therapy is increasing recently, their impact on fertility should be addressed in future research.

### Limitations

We are aware of the shortcomings and limitations of the present study. Even though the initial number of considered sperm banking patients was high (1089 patients), the survey had a relatively small number of participants and reported sperm usages. Due to the low response rate of 21.5%, participation bias may have an influence on our findings. It is not possible to predict how these outcomes would have differed with a larger study cohort. Further limitations are found in the lack of information about semen characteristics after treatment, details on ART procedures, and pregnancy attempts. The study also has a retrospective design, our questionnaire was not validated previously, and most of the information was self-reported by the patients. Due to these limitations, our study findings should be interpreted with caution and might be more indicative than generalizable.

### Implications

The findings presented in this study affirm that the cryopreservation of sperm is an effective fertility preservation method for male patients treated with gonadotoxic therapies and that more than half of the patients achieve parenthood by using the cryopreserved material. Our results may serve a practical application for health care providers and their patients when discussing the possible benefits of fertility preservation programs. Moreover, our data indicates that treatments with high gonadotoxicity might represent a predictive factor for future sperm usage and may encourage patients to store reproductive material, especially before undergoing treatments with a high risk for fertility loss. Given that the group of sperm users reported in this study consisted of oncological as well as non-oncological patients, the results emphasize the importance that cryopreservation should be offered to all patients before initiating a fertility-damaging therapy.

## Data Availability Statement

The original contributions presented in the study are included in the article/**Supplementary Material**. Further inquiries can be directed to the corresponding author.

## Ethics Statement

The studies involving human participants were reviewed and approved by Ethics Committee of Charité - Universitätsmedizin Berlin, corporate member of Freie Universität Berlin and Humboldt-Universität zu Berlin. The patients/participants provided their written informed consent to participate in this study.

## Author Contributions

PC conceived of the present idea and supervised the whole study. NL and PC contributed to conception and design of the study. IW provided the initial list of possible study participants and semen analyses for included patients. IJ, UK, LB, and SS were involved in treatments of study patients. NL performed the study preparation, data collection and evaluation as well as the statistical analysis of the data. NL wrote the manuscript in consultation with PC. All authors contributed to manuscript revision, provided critical feedback, read, and approved the submitted version.

## Funding

The authors received funds from Charité – Universitätsmedizin Berlin, corporate member of Freie Universität Berlin and Humboldt-Universität zu Berlin (Department of Hematology, Oncology, and Tumor Immunology, and Charité Medical Library).

## Conflict of Interest

The authors declare that the research was conducted in the absence of any commercial or financial relationships that could be construed as a potential conflict of interest.

## Publisher’s Note

All claims expressed in this article are solely those of the authors and do not necessarily represent those of their affiliated organizations, or those of the publisher, the editors and the reviewers. Any product that may be evaluated in this article, or claim that may be made by its manufacturer, is not guaranteed or endorsed by the publisher.

## Abbreviations

ART, assisted reproductive techniques. HSCT, hematopoietic stem cell transplantation; TBI, total body irradiation; IQR, interquartile range.
