# Supplementary material for: Survey of Long-Term Experiences of Sperm Cryopreservation in Oncological and Non-Oncological Patients: Usage and Reproductive Outcomes of a Large Monocentric Cohort
Source: Front Oncol. 2021 Nov 5;11:772809. doi: 10.3389/fonc.2021.772809 (PMC8602360; doi:10.3389/fonc.2021.772809)
Supplement: Supplementary file 1 [file DataSheet_1.pdf]

CharitéCentrum für Tumormedizin

Charité | Campus Mitte | 10117 Berlin

Medizinische Klinik mit Schwerpunkt
Onkologie, Hämatologie und Tumorimmunologie (CCM)

Standort Charité Campus Mitte
Direktor der Klinik: Prof. Dr. med. Sebastian Stintzing

Studienarzt: Prof. Dr. med. Philipp le Coutre
Tel.: (030) 450 665 307
E-Mail: philipp.lecoutre@charite.de

Doktorandin: Nadine Lackamp
E-Mail: nadine.lackamp@charite.de

English translation of the study questionnaire:

Fragebogen für Patienten *(Questionnaire for patients)*

1. Fragen zu Ihrer Person *(Personal data)*

Name: ______________________________
(Name)
Geburtsdatum: ______________________________
(Date of birth)
Adresse: ______________________________
(Mailing address) ______________________________
Telefon: ______________________________
(Phone number)
E-Mail: ______________________________
(E-Mail address)
Beruf: ______________________________
(Occupation)

2. Fragen zur Spermienkonservierung (Questions related to sperm cryopreservation)

2.1 Haben Sie in der Vergangenheit Spermien konservieren lassen?
(Did you cryopreserve sperm in the past?)

☐ ja, Datum/Daten *(Yes, date/s)*: ______________________________

☐ nein (falls zutreffend bitte direkt zu 3.) *(No)*

2.2 Wie häufig haben Sie Spermien einfrieren lassen?
(How often did you cryopreserve sperm?)

Name: ______________________________________

☐ 1x ☐ mehrmals *(more than once)*

2.3 Was war der Grund der Spermienkonservierung?

(What was the reason for the sperm cryopreservation?)

☐ Chemotherapie (falls zutreffend bitte 2.3.1 angeben) *(Chemotherapy)*

☐ Bestrahlung (falls zutreffend bitte 2.3.2 angeben) *(Radiotherapy)*

☐ Stammzelltransplantation (falls zutreffend bitte 2.3.3 angeben) *(Stem cell transplantation)*

☐ Hodenentfernung (falls zutreffend bitte 2.3.4 und 2.3.5 angeben) *(Orchiectomy)*

☐ andere Operation: ________________________________ (falls zutreffend bitte 2.3.6 angeben) *(Other surgery)*

☐ Kinderwunsch bei Unfruchtbarkeit (falls zutreffend bitte weiter zu 2.4) *(Infertility and wish to have a child)*

☐ sonstiger Grund: ________________________________ (falls zutreffend bitte weiter zu 2.4) *(Other reason)*

2.3.1 Wann war die Spermienkonservierung in Bezug auf die Chemotherapie?
(When was the sperm cryopreservation in relation to the chemotherapy?)

☐ vor Chemotherapie *(Before chemotherapy)* ☐ während der Chemotherapie *(During chemotherapy)* ☐ nach Beendigung der Chemotherapie *(After chemotherapy)*

☐ Datum der 1. Chemotherapie (falls erinnerlich): ______________________ *(Date of 1st chemotherapy)*

2.3.2 Wann war die Spermienkonservierung in Bezug auf die Bestrahlung?
(When was the sperm cryopreservation in relation to the radiotherapy?)

☐ vor Bestrahlung *(Before radiotherapy)* ☐ während der Bestrahlung *(During radiotherapy)* ☐ nach Beendigung der Bestrahlung *(After radiotherapy)*

☐ Datum der 1. Bestrahlung (falls erinnerlich): ______________________ *(Date of 1st radiotherapy)*

2.3.3 Wann war die Spermienkonservierung in Bezug auf die Stammzelltransplantation?
(When was the sperm cryopreservation in relation to the stem cell transplantation?)

☐ vor Stammzelltransplantation *(Before stem cell transplantation)* ☐ nach Stammzelltransplantation *(After stem cell transplantation)*

☐ Datum der Stammzelltransplantation (falls erinnerlich): ______________________ *(Date of stem cell transplantation)*

Name: ____________________________________

2.3.4 Wann war die Spermienkonservierung in Bezug auf die Hodenoperation?
(When was the sperm cryopreservation in relation to the orchiectomy?)

☐ vor Hodenoperation *(Before orchiectomy)* ☐ nach Hodenoperation *(After orchiectomy)*

☐ Datum der Hodenoperation (falls erinnerlich): ____________________ *(Date of orchiectomy)*

2.3.5 Welcher Hoden wurde entfernt? *(Which testicle was removed?)*

☐ Hodenentfernung links *(Left orchiectomy)* ☐ Hodenentfernung rechts *(Right orchiectomy)*

☐ Hodenentfernung beidseits *(Orchiectomy on both sides)*

2.3.6 Wann war die Spermienkonservierung in Bezug auf die Operation?
(When was the sperm cryopreservation in relation to the surgery?)

☐ vor Operation *(Before surgery)* ☐ nach Operation *(After surgery)*

☐ Datum der Operation (falls erinnerlich): ____________________ *(Date of surgery)*

2.4 Haben Sie Ihre konservierten Spermien zu einem späteren Zeitpunkt abgeholt?
(Did you request your cryopreserved sperm afterwards?)

☐ ja, Datum der Abholung: ____________________ *(Yes, date of the request)*

2.4.1 Wurden mit den Spermien Kinder gezeugt? *(Did you father children with the sperm?)*

☐ ja, Anzahl: ______ *(Yes, number of children)* ☐ nein *(No)*

☐ Versuch leider erfolglos *(Attempts remained unsuccessful)*

☐ nein, Spermien wurden nicht abgeholt *(No, I did not request my sperm)*

☐ Nutzung der Spermien nicht geplant *(Usage of sperm not planned)*

☐ Nutzung der Spermien in Zukunft geplant *(Usage of sperm planned in the future)*

3. Fragen zur Krankengeschichte *(Questions related to medical history)*

3.1 Haben Sie aufgrund einer Erkrankung oder deren Therapie Ihre Spermien einfrieren lassen? *(Did you undergo sperm cryopreservation because of a disease or disease treatment?)*

☐ ja *(Yes)* ☐ nein (falls zutreffend bitte direkt zu 4.) *(No)*

Name: ______________________________________

3.2 Bitte kreuzen Sie die für die Spermieneinfrierung relevante Erkrankung an.
(Please specify the relevant disease.)

☐ Hodenkrebs *(Testicular cancer)*

☐ Leukämie *(Leukemia)*

☐ Akute myeloische Leukämie *(Acute myeloid leukemia)* ☐ Chronische myeloische Leukämie *(Chronic myeloid leukemia)*

☐ Akute lymphatische Leukämie *(Acute lymphocytic leukemia)*

☐ Chronische lymphatische Leukämie *(Chronic lymphocytic leukemia)*

☐ Lymphom *(Lymphoma)*

☐ Morbus Hodgkin *(Hodgkin's lymphoma)* ☐ B-Zell Non-Hodgkin Lymphom *(B-cell non-Hodgkin lymphoma)*

☐ T-Zell Non-Hodgkin Lymphom *(T-cell non-Hodgkin lymphoma)*

☐ Burkitt-Lymphom *(Burkitt lymphoma)*

☐ andere Art des Lymphoms *(Other type of lymphoma)*

☐ Darmkrebs *(Gastrointestinal cancer)*

☐ Sarkom, Art des Sarkoms: ____________________________________ *(Sarcoma, type of sarcoma)*

☐ andere Krebserkrankung: ____________________________________ *(Other type of cancer)*

☐ andere Erkrankung: ____ *(Other disease)*

3.3 Wann wurde die Erkrankung zum ersten Mal diagnostiziert?
(When was the disease first diagnosed?)

Monat/Jahr: _________________ *(Month/year)*

3.4 Mit welcher Therapie/Therapien wurde die Erkrankung behandelt?
(Which treatment/s did you receive?)

☐ Chemotherapie, verwendete Chemotherapeutika: *(Chemotherapy, used agents)*

____

____

☐ Operation: ____ *(Surgery)*

☐ Bestrahlung *(Radiotherapy)*

☐ Ganzkörperbestrahlung *(Total body irradiation)* ☐ lokale Bestrahlung *(Local irradiation)*

Name: ______________________________________

☐ andere Therapie: ____ *(Other treatment)*

3.5 Ist die Erkrankung nach der ersten Therapie erneut aufgetreten?
(Did you suffer from disease relapses?)

☐ ja, Monat/Jahr des Rezidivs: ________________ *(Yes, month/year of the relapse)*

☐ nein *(No)*

3.6 Leiden Sie noch heute an dieser Erkrankung?
(Do you suffer from the disease currently?)

☐ ja *(Yes)* ☐ nein *(No)*

4. Fragen zur Familie *(Questions related to the familiy)*

4.1 Familienstand *(Familiy status)*

☐ ledig *(Single)* ☐ in Partnerschaft lebend *(In partnership)*

☐ verheiratet *(Married)* ☐ verwitwet *(Widowed)* ☐ anderes *(Other)*

4.2 Haben Sie Kinder? *(Do you have children?)*

☐ ja, Anzahl: _________ *(Yes, number)* ☐ Partnerin schwanger *(Pregnant partner)*

☐ nein *(No)*

4.3 Bitte füllen Sie die Tabelle bezüglich Ihrer Kinder aus (bei mehr als 4 Kindern bitte Tabelle auf Rückseite des Fragebogens erweitern).
(Please complete the following table.)

	**Kind 1 *(Child 1)***	**Kind 2 *(Child 2)***	**Kind 3 *(Child 3)***	**Kind 4 *(Child 4)***
**Geburtsdatum *(Date of birth)***				
**Geschlecht *(Gender)***	☐ männlich *(Male)*<br>☐ weiblich *(Female)*<br>☐ divers *(Non-binary)*	☐ männlich *(Male)*<br>☐ weiblich *(Female)*<br>☐ divers *(Non-binary)*	☐ männlich *(Male)*<br>☐ weiblich *(Female)*<br>☐ divers *(Non-binary)*	☐ männlich *(Male)*<br>☐ weiblich *(Female)*<br>☐ divers *(Non-binary)*
**Leiblich/nicht**	☐ leiblich	☐ leiblich	☐ leiblich	☐ leiblich

Name: ______________________________________

**leiblich** **(*Biological/not biological*)**	*(Biological)* ☐ nicht leiblich *(Not biological)*	*(Biological)* ☐ nicht leiblich *(Not biological)*	*(Biological)* ☐ nicht leiblich *(Not biological)*	*(Biological)* ☐ nicht leiblich *(Not biological)*
**Art der Zeugung des Kindes *(Type of reproduction)***	☐ natürlich *(Natural)*<br>☐ künstliche Befruchtung mit konservierten Spermien *(Fertilization with cryopreserved sperm)*<br>☐ andere *(Other)*	☐ natürlich *(Natural)*<br>☐ künstliche Befruchtung mit konservierten Spermien *(Fertilization with cryopreserved sperm)*<br>☐ andere *(Other)*	☐ natürlich *(Natural)*<br>☐ künstliche Befruchtung mit konservierten Spermien *(Fertilization with cryopreserved sperm)*<br>☐ andere *(Other)*	☐ natürlich *(Natural)*<br>☐ künstliche Befruchtung mit konservierten Spermien *(Fertilization with cryopreserved sperm)*<br>☐ andere *(Other)*
**Geburtsgewicht *(Birth weight)***	________ g	________ g	________ g	________ g
**Geburtsgröße *(Height at birth)***	________ cm	________ cm	________ cm	________ cm
**Komplikationen während der Geburt *(Events during birth)***	☐ nein *(No)*<br>☐ ja *(Yes)*	☐ nein *(No)*<br>☐ ja *(Yes)*	☐ nein *(No)*<br>☐ ja *(Yes)*	☐ nein *(No)*<br>☐ ja *(Yes)*
**Gesundheitszustand des Kindes heute *(Current health status of the child)***	☐ gesund, normal entwickelt *(Healthy, normally developing)*<br>☐ erkrankt *(Diseased)*: ____________<br>____________<br>____________<br>☐ verstorben *(Deceased)*	☐ gesund, normal entwickelt *(Healthy, normally developing)*<br>☐ erkrankt *(Diseased)*: ____________<br>____________<br>____________<br>☐ verstorben *(Deceased)*	☐ gesund, normal entwickelt *(Healthy, normally developing)*<br>☐ erkrankt *(Diseased)*: ____________<br>____________<br>____________<br>☐ verstorben *(Deceased)*	☐ gesund, normal entwickelt *(Healthy, normally developing)*<br>☐ erkrankt *(Diseased)*: ____________<br>____________<br>____________<br>☐ verstorben *(Deceased)*
**Geburtsdatum der Mutter *(Date of birth of mother)***				

Vielen Dank für Ihre Teilnahme!
